# Impact of Polypyridyl Ru Complexes on Angiogenesis—Contribution to Their Antimetastatic Activity

**DOI:** 10.3390/ijms23147708

**Published:** 2022-07-12

**Authors:** Ilona Gurgul, Olga Mazuryk, Kamila Stachyra, Rafał Olszanecki, Małgorzata Lekka, Michał Łomzik, Franck Suzenet, Philippe C. Gros, Małgorzata Brindell

**Affiliations:** 1 Faculty of Chemistry, Jagiellonian University in Krakow, Gronostajowa 2, 30-387 Krakow, Poland; ilona.gurgul@uj.edu.pl (I.G.); michal.lomzik@chemia.uni.lodz.pl (M.Ł.); 2 Chair of Pharmacology, Faculty of Medicine, Jagiellonian University Medical College, Grzegorzecka 16, 31-531 Krakow, Poland; kamila.stachyra@uj.edu.pl (K.S.); rafal.olszanecki@uj.edu.pl (R.O.); 3 Department of Biophysical Microstructures, Institute of Nuclear Physics, Polish Academy of Sciences, PL-31342 Krakow, Poland; malgorzata.lekka@ifj.edu.pl; 4 Department of Organic Chemistry, Faculty of Chemistry, University of Łódź, ul. Tamka 12, 91-403 Łódź, Poland; 5 Institute of Organic and Analytical Chemistry, University of Orléans, UMR-CNRS 7311, rue de Chartres, BP 6759, CEDEX 2, 45067 Orléans, France; franck.suzenet@univ-orleans.fr; 6 Université de Lorraine, CNRS, L2CM, F-54000 Nancy, France; philippe.gros@univ-lorraine.fr

**Keywords:** polypyridyl ruthenium (II) complexes, endothelial cells, angiogenesis, cell adhesion properties, cytotoxicity, migration, cell elasticity, focal adhesions, pseudovessel formation

## Abstract

The use of polypyridyl Ru complexes to inhibit metastasis is a novel approach, and recent studies have shown promising results. We have reported recently that Ru (II) complexes gathering two 4,7-diphenyl-1,10-phenanthroline (dip) ligands and the one being 2,2′-bipyridine (bpy) or its derivative with a 4-[3-(2-nitro-1H-imidazol-1-yl)propyl (bpy-NitroIm) or 5-(4-{4′-methyl-[2,2′-bipyridine]-4-yl}but-1-yn-1-yl)pyridine-2-carbaldehyde semicarbazone (bpy-SC) moieties can alter the metastatic cascade, among others, by modulating cell adhesion properties. In this work, we show further studies of this group of complexes by evaluating their effect on HMEC-1 endothelial cells. While all the tested complexes significantly inhibited the endothelial cell migration, Ru-bpy additionally interrupted the pseudovessels formation. Functional changes in endothelial cells might arise from the impact of the studied compounds on cell elasticity and expression of proteins (vinculin and paxillin) involved in focal adhesions. Furthermore, molecular studies showed that complexes modulate the expression of cell adhesion molecules, which has been suggested to be one of the factors that mediate the activation of angiogenesis. Based on the performed studies, we can conclude that the investigated polypyridyl Ru (II) complexes can deregulate the functionality of endothelial cells which may lead to the inhibition of angiogenesis.

## 1. Introduction

**Figure 1:**
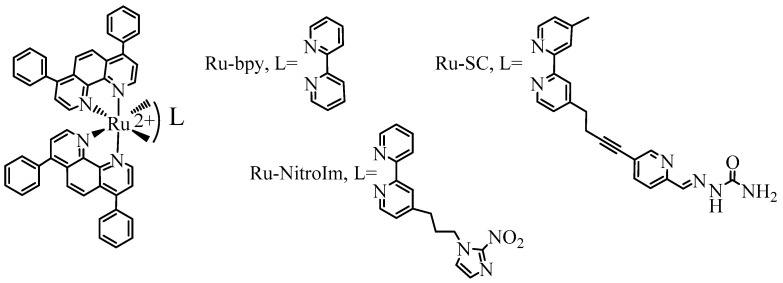
The studied ruthenium (II) polypyridyl complexes.

Recently, polypyridyl Ru complexes deserved particular attention from researchers searching for novel anticancer agents due to their high stability arising from their inertness to substitution and oxidation [1]. Furthermore, by appropriate design of the ligands, they can reach selected targets [2,3], or their activity can be triggered by various stimuli such as redox, pH, enzyme, and light, among others [4]. Most studies focused on elucidating the mechanism of activity related to their cytotoxicity; however, the main problem in cancer treatment is the inhibition of metastasis development. The use of metal complexes to inhibit metastasis is a relatively new approach [5,6], and the first metal complex with antimetastatic properties in clinical trials was (ImH) [*trans*-RuCl_4_(DMSO)(Im)] (where Im is imidazole), the so-called NAMI-A [5,7]. It affected many stages of metastasis, including the angiogenesis process [8,9]. New attractive candidates for antimetastatic agents are polypyridyl Ru complexes, which can affect various metastatic hallmarks such as cell migration, invasion, and adhesion [6,10,11,12,13,14]. For the Ru compounds studied in this work (Figure 1), we have shown that the observed inhibition of cancer cell motility and reinforcement of their adhesion arise from their impact on the activity of the selected integrins and upregulation of the expression of focal adhesion components such as vinculin and paxillin [14]. Studies on the impact of polypyridyl Ru complexes on endothelial cells, the angiogenesis process, and molecular targets related to this process are among the few. Among others, the treatment with Ru complexes was shown to result in a reduction in the bioavailability of vascular endothelial growth factor (VEGF) [11], affecting pseudo vessels formation in vitro [10], and inhibition of angiogenesis in an in vivo model of zebrafish [12].

Angiogenesis is the process of the formation of new blood vessels from existing ones. It is of great importance in the pathophysiology of many diseases. Among others, it is essential for sustained tumor growth and the hematogenous spreading of cancer cells [15]. Most do not reach a volume greater than 2–3 mm^3^ without creating a blood vessel network. The presence of blood vessels within the tumor allows not only oxygen and nutrients transport and the removal of metabolic products but also the penetration of cancer cells into the bloodstream; hence the creation of metastasis. The occurrence of such a complex process requires the cooperation of many cells, cytokines, and the extracellular matrix simultaneously. In the initiation stage, an increase in the permeability of blood vessels and the accumulation of extracellular proteins in the extracellular matrix (ECM) are characteristic. Following degradation of the components of the basal membranes, as well as vessel wall reorganization and proteolysis. When all these events occur together, invasion, migration, and proliferation of endothelial cells are possible [16]. The alteration of endothelial cell adhesion properties and the impact on the ability to form focal adhesion sites are related to their migration and the ability to form pseudovessels [17]; however, it is not the only process in which adhesion molecules are involved. Adhesion molecules are crucial in modulating interactions between cells. Vascular cell adhesion molecule 1 (VCAM-1) is present in endothelial cells, increasing its expression under the influence of pro-inflammatory cytokines. It is postulated that the binding of VCAM-1 to integrins mediates the adhesion of cancer cells to endothelial cells, allowing the extravasation process [18].

Angiogenesis is considered a promising therapeutic target in cancer treatment [19,20,21]. Drugs acting as angiogenesis inhibitors have already been approved for use in anticancer therapies [22]. Most compounds work indirectly by removing angiogenic growth factors from circulation or blocking their receptors or signaling pathways. Another group of angiogenesis inhibitors is composed of compounds that act directly on the endothelium and affect cellular regulatory pathways independent of cancer cells. Such therapy by direct targeting of endothelial cells is likely to reduce the risk of developing drug resistance [23]. Metalloproteinases (MMPs) play a crucial role in the angiogenesis process. These enzymes are involved in extracellular matrix degradation and influence endothelial cell adhesion properties [24]. MMPs induce the initiation of angiogenesis in both physiological and pathological processes. They also have a decisive impact on the activation of pro-angiogenic, and in some cases, antiangiogenic factors in cancer tissues. Thus, MMPs can be considered angiomodulators, which can control the formation of new vessels necessary for cancer growth, progression, and spread [25]. MMPs inhibitors such as Marimastat or Prinomastat have entered the third phase of clinical trials as potential antiangiogenic drugs [22]. We previously reported that two polypyridyl Ru complexes, Ru-SC and Ru-bpy, having structures shown in Figure 1, are potent inhibitors of MMP2 and MMP9 and reduced MMP9 expression levels in lung cancer cells lysates [13,26], whereas Ru-bpy and Ru-NitroIm (structure shown in Figure 1) decreased the expression level of MMP9 mRNA under hypoxic conditions [10]. MMP2 and MMP9 participate in angiogenesis by remodeling ECM, activating and deactivating their components by proteolysis cleavage. It results, among others, in promoting endothelial cell migration and triggering the angiogenic switch [25].

In this work, we have investigated the impact of a series of Ru (II) polypyridyl complexes of the type [Ru(dip)_2_L]^2+^ (L = bpy, bpy-SC, bpy-NitroIm; depicted in Figure 1) on the HMEC-1 endothelial cell. Their ability to promote pseudovessels formation and migration of endothelial cells has been evaluated. Furthermore, changes in cell elasticity and the expression of proteins involved in focal adhesions that are closely related to cell migration properties are investigated. The impact of treatment of endothelial cells with Ru complexes on the expression of cell adhesion molecule 1 (VCAM-1) and intercellular adhesion molecule 1 (ICAM-1) involved, among others, in leukocyte recruitment was also checked.

## 2. Results and Discussion

The conducted research on the impact of Ru complexes on angiogenesis requires the use of non-toxic doses of compounds to provide information about functional changes in living cells induced by the studied Ru compounds. All studies were carried out on the dermal microvascular endothelial cells (HMEC-1), and their viability after treatment was investigated using resazurin dye (Alamar Blue test) that is based on the reducing power of living cells. The studied Ru complexes inhibited the growth of HMEC-1 cells in a dose-dependent manner with an IC_50_ parameter of approximately 5–7 µM (Table 1). Therefore, these types of complexes are quite cytotoxic to endothelial cells. The same level of cytotoxicity was found for various cancer cell lines [10,13,26], indicating that these compounds are not selective. Thus, an appropriately low dose has to be applied to avoid damage to healthy cells.

### 2.1. Negative Impact on Tube Formation

**Figure 2:**
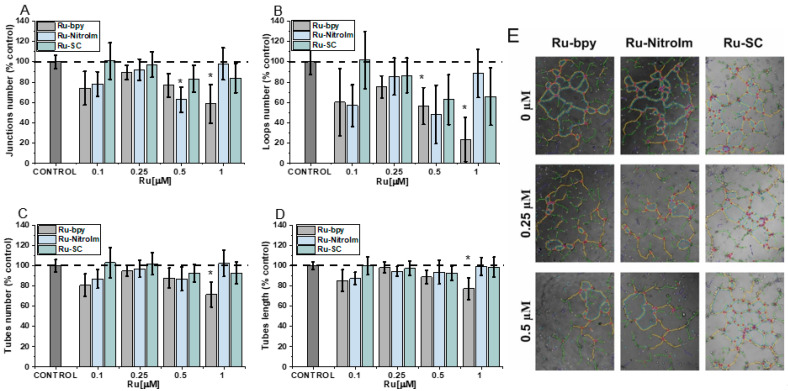
The influence of [Ru(dip)_2_(bpy)]Cl_2_ (Ru-bpy, gray), [Ru(dip)_2_(bpy-NitroIm)]Cl_2_ (Ru-NitroIm, blue) and Ru(dip)_2_(bpy-SC)]Cl_2_ (Ru-SC, green) on the angiogenesis process in HMEC-1 endothelial cells, expressed as (**A**) a number of junctions, (**B**) a number of loops, (**C**) a number of tubes, (**D**) a length of tubes and (**E**) microscopic images after analysis in ImageJ. The results were calculated relative to the control cells (100%). * *p* < 0.05.

The fundamental step in the formation of new vessels is the arrangement of many cells in tubular aggregates, which is possible due to the three-dimensional structure of the extracellular matrix (ECM) and the contact of its components with endothelial cells. The influence of the studied compounds on angiogenesis was evaluated using the tube formation assay, which has already been implemented for the other Ru polypyridyl complexes [6,10,12]. In this assay, endothelial cells are seeded on a basement-membrane-like substrate, on which the cells can form tubules [6]. During the analysis of pseudovessel formation by HMEC-1 cells, parameters such as the number and length of tubes as well as the number of branching points and loops were quantified (Figure 2). The greatest effect on the formation of pseudoveasels was observed for Ru-bpy, the complex having bpy ligand without any substituent. A higher concentration (1 µM) caused a statistically significant decrease in the number of branching points, loops, tubes and reduced their total length (Figure 2). The reduction in the number of loops was quite extended (Figure 2C), indicating that after Ru-bpy treatment, cells create a less organized tube network than controlled cells. Ru-NitroIm at 0.5 µM concentration also negatively influenced tube formation, manifested in a smaller number of loops and junctions (Figure 2A,B). Disruption of tube formation was also recognized for antimetastatic NAMI-A [9] as well as for clinically tested angiogenesis inhibitors such as bevacizumab [27] or sunitinib [28]. The tube formation assay encompasses many processes such as proliferation, differentiation, migration, apoptosis, etc. which need to cooperate to successfully reorganized endothelial cells into three-dimensional tubular structures. To further interrogate these processes, we investigated the changes in cell migration induced by the studied Ru complexes.

### 2.2. Inhibitory Effect on Endothelial Cell Migration

**Figure 3:**
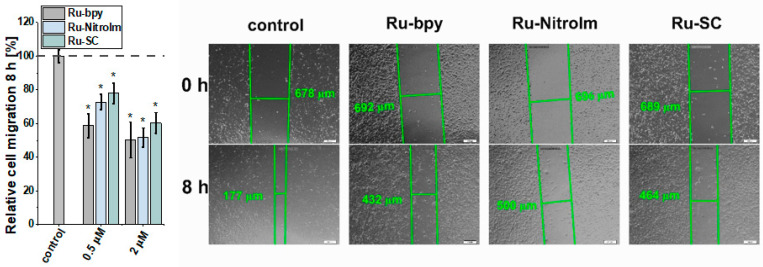
Influence of [Ru(dip)_2_(bpy)]Cl_2_ (Ru-bpy, gray), [Ru(dip)_2_(bpy-NitroIm)]Cl_2_ (Ru-NitroIm, blue) and Ru(dip)_2_(bpy-SC)]Cl_2_ (Ru-SC, green) (0.5 or 2 µM) on HMEC-1 cells migration determined using a wound healing assay after 8 h. Untreated cells were used as a control (100%). Bars represent the relative cell migration measured as a difference in wound width at zero time and after 8 h of incubation in relation to control. * *p* < 0.05.

The migration of endothelial cells is a prerequisite for the growth of new capillaries. They might be recruited far away from the existing vascular bed by the production of a chemotactic gradient composed of growth factors secreted by the tumor [29]. Therefore, the influence of Ru complexes on the migration ability of HMEC-1 cells was investigated by a wound healing (scratch) assay, which involves the creation of an artificial gap (cell-free area) in a monolayer of endothelial cells and subsequent measurement of the recovered area with migrating cells. All tested complexes significantly inhibited endothelial cell migration as determined by changes in the scratch area after 8 h (Figure 3). The most significant effect was observed for the parent compound Ru-bpy, which showed inhibition of cell migration by ca. 58% compared to the control at as low a dosage as 0.5 µM. These observations correlate well with the most significant influence of Ru-bpy on the disorganization of pseudo-vessel formation. At a higher dosage of 2 µM, all compounds inhibited the migration of cells quite well. Our previous studies conducted on endothelial MLuMEC FVB cells also showed the negative impact of Ru-NitroIm on their motility [10]. Most studies on the influence of Ru polypyridyl complexes on cell mobility were performed on cancer cells [6], and in many cases, a significant inhibition of migration was noted. We recently have also shown the inhibitory properties of Ru complexes studied in this work on several cancer cell lines [14]. Therefore, the molecular mechanism responsible for the observed effect on endothelial and cancer cells might be similar. In particular, the effect on cell mobility was also evident in NAMI-A [9] and the clinically tested angiogenesis inhibitors [27,28]. Changes in cell motility were recognized as one of the crucial factors related to the observed anti-angiogenic activity of drugs. Thus, we further explored the impact of the Ru complexes on cell elasticity and focal adhesion components that are closely related to cell motility.

### 2.3. Impact on Cell Elasticity and Focal Adhesion Components

**Figure 4:**
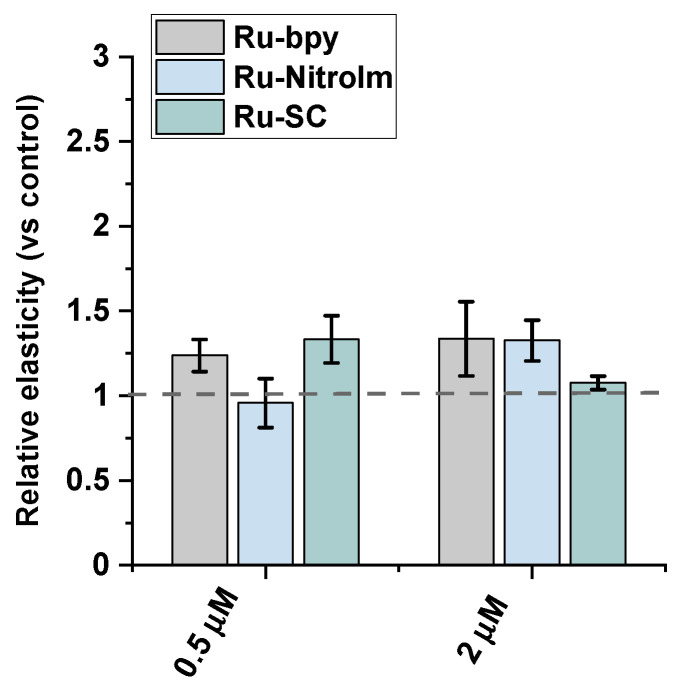
AFM nanomechanical measurements of elasticity presented as a change in Young’s modulus (kPa) of HMEC-1 cells after 24 h treatment with [Ru(dip)_2_(bpy)]Cl_2_ (gray), [Ru(dip)_2_(bpy-nitro)]Cl_2_ (blue) and Ru(dip)_2_(bpy-SC)]Cl_2_ (green) (0.5 or 2 µM). The results were calculated by analyzing ~60 randomly selected Ru-treated adherent cells in respect to control (untreated) cells (dashed line).

Endothelial cell migration is mediated by remodeling the actin cytoskeleton and is linked to focal adhesions that anchor cells to the substrate, mainly the extracellular matrix. Recently, a correlation between cell elasticity, a vital mechanical property of cells, and cell motility has been widely reported. Cell stiffness is described as Young’s modulus (or elastic modulus) obtained prevailingly by atomic force microscopy (AFM) measurements. Stiffness is determined by probing the cantilever into the cell surface (usually in the central/nuclear region to record global stiffness), and the indentation depth and deflection of the cantilever are recorded [30]. Several studies showed that cancerous cells are softer than their benign counterparts [30,31,32]. Softer cells usually have higher motility, since being softer is an advantage in migration and invasion [30]. To check whether the lower motility of Ru-treated endothelial cells is correlated with a change in their mechanical properties, Young’s modulus of Ru-treated and untreated HMEC-1 cells was measured using AFM by probing cells in their central region (nucleus) (Figure 4). AFM studies revealed that polypyridyl ruthenium (II) complexes increased Young’s moduli of the cells indicating a greater rigidity of endothelial cells after Ru treatment. A similar effect was observed in the case of the highly metastatic breast cancer cell line MDA-MB-231 [14]. The increased rigidity of endothelial cells correlated well with the decrease in motility of HMEC-1 in a wound healing assay (Figure 3).

**Figure 5:**
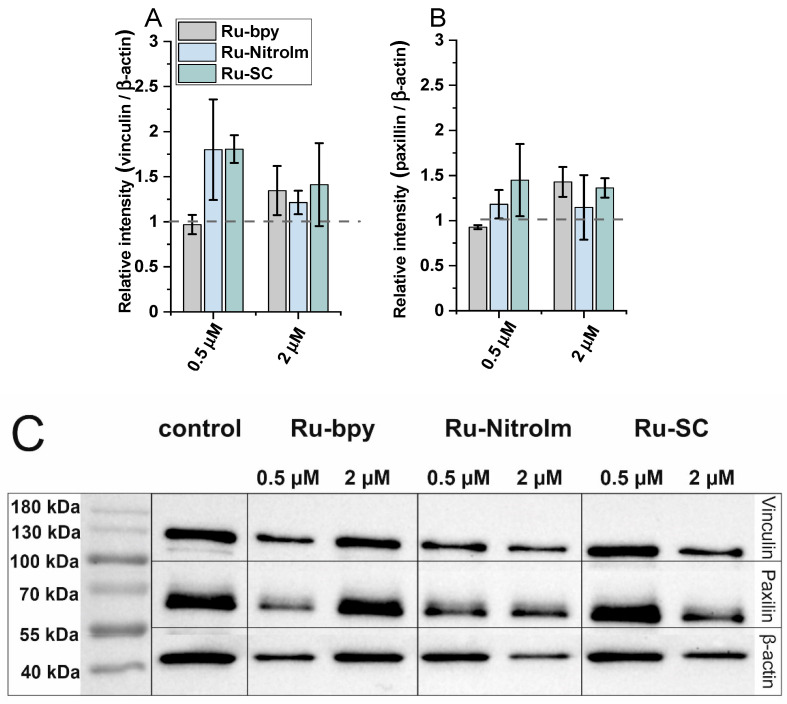
The expression levels of (**A**) vinculin and (**B**) paxillin measured by (**C**) western blot in HMEC-1 protein extracts obtained after 24 h of treatment with [Ru(dip)_2_(bpy)]Cl_2_ (gray), [Ru(dip)_2_(bpy-nitro)]Cl_2_ (blue) and Ru(dip)_2_(bpy-SC)]Cl_2_ (green) (0.5 or 2 µM). Expression for vinculin and paxillin were calculated with respect to β-actin.

In addition, since the expression of focal adhesion components, such as vinculin and paxillin, might be related to the inhibition of migration and the ability to form pseudovessels by endothelial cells [17,33], the impact of the studied complexes on the expression levels of vinculin and paxillin has been tested by applying western blot (Figure 5C). Both proteins are involved in focal adhesions, and vinculin serves as a structural protein while paxillin has a signaling role. As shown in Figure 5A, a significant increase in the amount of vinculin was observed in the protein extracts of HMEC-1 cells previously treated with Ru-SC at a low concentration (0.5 µM). The treatment of cells with 2 µM of all studied compounds induced a slight increase in the expression of vinculin (Figure 5A). It was recently shown that inhibiting of vinculin expression in endothelial cells promotes angiogenesis [17], while the increased expression of this protein in tumor endothelial cells was related to the inhibition of their migration. Thus, the observed increase in vinculin concentration might contribute to the inhibitory effect of Ru complexes on HMEC-1 cell migration. Furthermore, the increase in the amount of paxillin upon treatment of HMEC-1 cells with both Ru-bpy and Ru-SC (Figure 5B) at a higher concentration (2 µM) was observed. Paxillin was shown to negatively regulate endothelial cell migration [33]. Therefore, the observed inhibition of HMEC-1 cell migration might at least partially arise from a positive impact of Ru complexes on the expression of this protein. The low effect on the expression of both proteins exhibited by Ru-bpy at low concentration (0.5 µM) compared to its high potency in endothelial cell migration and tube formation inhibition indicates that these proteins are not their key targets. Other mechanisms might be involved in their antiangiogenic activity, and further studies are needed to find the molecular targets for the studied compounds.

### 2.4. Effect on Endothelial Cells Response to TNF-α Cytokine

One of the crucial functions of endothelial cells is the recruitment of leukocytes in response to some inflammatory stimulations. Intercellular adhesion molecule 1 (ICAM-1) and vascular cell adhesion molecule 1 (VCAM-1) are endothelial cell adhesion molecules (CAMs). They are crucial in mediating endothelial cell-leucocyte interactions leading to their firm adhesion to the endothelium and transendothelial migration [34]. Expression of both VCAM-1 and ICAM-1 increases under the influence of pro-inflammatory cytokines. Therefore, to evaluate the impact of Ru complexes on the expression of VCAM-1 and ICAM-1, endothelial cells were treated with tumor necrosis factor-*α* (TNF-*α*), an inflammatory cytokine. The western blot technique was used to evaluate the effect of the studied complexes on VCAM-1 and ICAM-1 expression levels in endothelial cell lysates.

**Figure 6:**
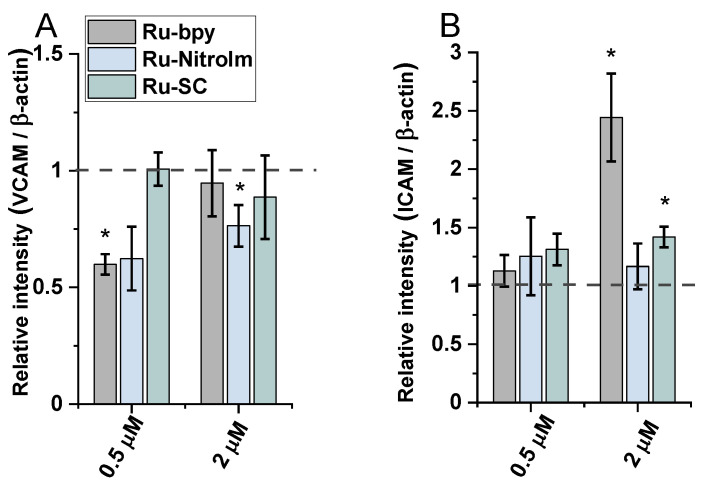
The expression levels of (**A**) VCAM-1 and (**B**) ICAM-1 measured by western blot in HMEC-1 protein extracts obtained after 24 h of treatment with [Ru(dip)_2_(bpy)]Cl_2_ (gray), [Ru(dip)_2_(bpy-nitro)]Cl_2_ (blue) and Ru(dip)_2_(bpy-SC)]Cl_2_ (green) (0.5 or 2 µM). TNF-α (0.1 ng/mL) was used as a positive inducer control. Expression for VCAM-1 and ICAM-1 were calculated with respect to β-actin. * *p* < 0.05.

The treatment of endothelial cells with Ru-bpy and Ru-NitroIm significantly reduced the level of VCAM-1 expression induced by TNF-α (Figure 6A), suggesting their negative impact on angiogenesis. It was proved that the microvessel density in gastric cancer tissues expressing VCAM-1 was significantly higher than in tissues not expressing this protein. Therefore, the expression level of VCAM-1 is suggested to be one of the factors that mediate angiogenesis activation [35]. Additionally, it was postulated that VCAM-1, by binding to α4β1 integrin, its major ligand, mediates the adhesion of cancer cells to endothelial cells, enabling their extravasation [36]. Integrin α4β1 is commonly known for its expression on leukocytes. However, its abnormal expression was also frequently observed on tumor cells leading to their transendothelial migration increase that contributes to metastasis [37,38]. Thus, decreasing the level of VCAM-1 expression by both complexes may reduce the likelihood of the adhesion of cancer cells to endothelial cells leading to the inhibition of the extravasation.

The treatment of endothelial cells activated by TNF-*α* with Ru complexes resulted in a significant rise in the expression of ICAM-1 in the case of Ru-bpy and a moderate increase for Ru-SC applied at a higher concentration (2 µM, Figure 6B). However, at a lower concentration of 0.5 µM, all three Ru complexes had quite a low effect on the expression of ICAM-1 compared to untreated cells. The increased expression of ICAM-1 on endothelial cells supports the transendothelial migration of cancer cells [39,40]. In some studies, it was shown that cancer cells are able to upregulate ICAM-1 expression on endothelial cells [41]. To avoid this undesired effect promoting metastasis, an appropriately low concentration of Ru-bpy should be applied.

Studies performed on the impact of Ru complexes on VCAM-1 and ICAM-1 show that the same molecules might be responsible for both desired and undesirable effects depending on the target they interact with and the applied concentration. Therefore, a comprehensive approach to in vitro studies and the careful interpretation of results are required.

## 3. Materials and Methods

### 3.1. Materials

All solvents were of analytical grade and were used without further purification. Reagents were purchased from Sigma-Aldrich. 2,2′-bipyridine (bpy) and 4,7-diphenyl-1,10-phenanthroline (dip) were purchased from Sigma-Aldrich. The following complexes [Ru(dip)_2_(bpy)]Cl_2_,[Ru(dip)_2_(bpy-SC)]Cl_2_ and [Ru(bpy)_2_bpy-NitroIm]Cl_2_ were prepared according to the published procedures [42,43,44]. The purity and identity were confirmed by HPLC by comparison to published results as well as ^1^H NMR or HRMS analysis (data available in Supplementary Materials).

### 3.2. Cell Culturing and Cytotoxicity Assay

The in vitro studies were conducted using a dermal microvascular endothelium HMEC-1 cell line. Cells were cultured in MCDB131 medium supplemented with 10 ng/mL epidermal growth factor (EGF), 1 µg/mL hydrocortisone, 10 mM glutamine, 10% fetal bovine serum (FBS) (*v*/*v*) and 1% penicillin-streptomycin solution (100 units/mL–100 µg/mL) (*v*/*v*) at 37 °C in humidified atmosphere with 5% CO_2_ (*v*/*v*). Cell viability upon treatment with Ru (II) complexes was determined using the Alamar Blue assay. Cells were seeded into a 96-well plate with the density of 3 × 10^4^ cells per cm^2^ in a complete medium and cultured for 24 h. Subsequently, cells were incubated with various concentrations of the studied complexes for 24 h. Stock solutions of the Ru (II) complexes were prepared in DMSO. The final DMSO concentration in cell culture was fixed at 0.1% (*v*/*v*). After 24 h of incubation, cells were washed with PBS and incubated in Alamar Blue solution for 3 h at 37 °C. Afterwards, the fluorescence was measured using a Tecan Infinite 200 microplate reader (Tecan Group Ltd., Männedorf, Switzerlan) at 605 nm using a 560 nm excitation light. Experiments were performed in triplicates and repeated three times. Results are presented as mean values and standard error of the mean. IC_50_ parameters were determined using the Hill equation (Origin 2020).

### 3.3. Tube-Formation Assay

HMEC-1 cells were seeded in a 96-well plate pre-coated with Geltrex^®^ LDEV-Free (40 μM/well, 1 h, 37 °C) at a density of 1.6 × 10^5^ cells per cm^2^, in a medium without FBS containing the appropriate concentration of the tested complex (DMSO content 0.1%). Cells were monitored every hour. Cell visualization was performed in a chamber integrated with the microscope ensuring appropriate conditions of temperature, humidity, and CO_2_ concentration (fluorescence microscope Olympus IX51, Olympus, Tokyo, Japan; and Incubation System cellVivo PECON, Ulm, Germany). For ImageJ Angiogenesis Analyzer [45,46] analysis, images collected after 11 h of incubation were chosen. The obtained results were calculated from three independent experiments, untreated cells were used as control.

### 3.4. Wound Healing Assay

Cells were seeded at a density of 1.6 × 10^5^ cells per cm^2^, 24 h before the experiments. A 200 µL pipette tip was used to scratch and create a wound in the monolayer of HMEC-1 cells. After washing with serum-free medium, the Ru complexes dissolved in serum free medium were added. The images showing migration of the cells into the free spaces were taken immediately after adding complexes and after 8 h using a fluorescence microscope (Olympus IX51). The presented results were calculated based on three independent experiments done in triplicate and wounds were measured in two different places for each one.

### 3.5. Atomic Force Microscopy—Elasticity Measurements

The biomechanical properties of HMEC-1 cells were evaluated by AFM using an XE-120 (Park System, Suwon, South Korea) with a combined Olympus IX71 inverted optical microscope (Olympus, Tokyo, Japan). The measurements were performed in a liquid environment in the basal cell culture medium at room temperature. Each experiment lasted no more than 2 h to preserve the cell viability. Measurements were performed using commercially available silicon nitride cantilevers with a nominal spring constant of 0.03 N/m, open-angle of 36° and tip radius of 10 nm (PNP-TR-B, Nanoworld). Prior to the experiments, the spring constant of the cantilever was measured using the thermal noise calibration.

HMEC-1 cells were seeded at a density of 8 × 10^3^ cells per cm^2^ in complete medium and incubated overnight to allow the cell to attach. Then cells were treated with 0.5 or 2 µM of Ru complexes for 24 h. After incubation, cells were washed with DPBS, and the coverslip with cells was transferred to a “liquid cell” placed on an AFM scanner. The cells were probed over the nuclear region of an individual cell. The 25 force curves were recorded over a scan area of 25 µm^2^. During each experiment, 15–20 cells were measured for each studied condition. The experiments were repeated three times. The force curves were converted into force versus indentation curves and further analyzed to calculate Young’s modulus. The results are presented as a ratio for the average values of Young’s modulus for Ru-treated cells and the untreated control.

### 3.6. Western-Blot Analysis

The expression levels of the proteins involved in focal adhesions (FAs), vinulin and paxillin, as well as ICAM-1 and VCAM-1 adhesion proteins in HMEC-1 cells were determined in cell lysates by western blot technique. After 24 h of incubation with the tested Ru (II) complexes, the cells were washed twice with PBS (4 °C) and lysed with a cold RIPA buffer. In the case of the determination of ICAM-1 and VCAM-1 in cells, prior to the addition of the complex solutions, were incubated for 15 min with the TNF-α solution (0.1 ng/mL). The lysates were centrifuged and the protein concentrations in the supernatant were determined by the Bradford method. Protein samples (the same concentration per lane) were separated on a sodium dodecyl sulfate polyacrylamide gel (12% for vinculin and paxillin. 7.5% for I-CAM and VCAM-1). Electrophoresis was performed using a PowerPac™ Basic Power Supply (Bio-Rad, Inc., Hercules, CA, USA). PageRuler™ Prestained Protein Ladder (Thermo Fisher Scientific, Waltham, NJ, USA) was used to determine the approximate molecular weights of resolved proteins. Proteins were then transferred from the gel to a polyvinylidene difluoride (PVDF) membrane (2 h/200 mA, Bio-Rad, Inc., Hercules, CA, USA) followed by blocking in 5% skim milk in TBST for 1 h (Trisbuffered Saline Tween-20). The membranes were incubated overnight at 4 °C with primary antibodies: mouse anti-paxillin monoclonal antibody (1:250 dilution; Thermo Fisher Scientific, Waltham, NJ, USA, Cat# AH00492); rabbit monoclonal anti-vinculin (1:500 dilution; Thermo Fisher Scientific, Waltham, NJ, USA, Cat# 42H89L44), mouse anti-β-actin monoclonal (1:1000 dilution; Thermo Fisher Scientific, Waltham, NJ, USA, Cat# AM4302), mouse monoclonal VCAM-1 (dilution 1:200; Santa Cruz Biotechnology, Dallas, TX, USA, Cat# sc-13160) and mouse monoclonal antibody ICAM-1 (dilution 1:200; Santa Cruz Biotechnology, Dallas, TX, USA, Cat# sc-8439). In the next step, the membranes were incubated for 2 h at room temperature with horseradish peroxidase labeled secondary antibodies. A goat antimouse antibody (dilution 1:10,000; Thermo Fisher Scientific, Waltham, NJ, USA, Cat# G21040) was used for the detection of paxillin/β-actin, and a goat anti-rabbit antibody (dilution 1:10,000; Thermo Fisher Scientific, Waltham, NJ, USA, Cat# G21234) was used for the detection of vinculin. The proteins examined were visualized with the use of a reagent for the detection of GE Healthcare Amersham™ ECL Prime Western Blotting (GE Healthcare Inc., Chicago, IL, USA). Data were collected on a ChemiDoc XRS + imaging system (Bio-Rad, Inc., Hercules, CA, USA) and analyzed using Image Lab v. Software 6.1.0 software (Bio-Rad, Inc., Hercules, CA, USA). The results were normalized to the intensity of β-actin.

### 3.7. Statistical Analysis

For in vitro experiments, all data were expressed as the mean and standard error of the mean (SEM). For statistical analysis between the control group and experiment groups, one-way analysis of variance (ANOVA) was performed, and the Mann-Whitney U test was applied for statistical analysis when the data didn’t accord with the homogeneity of variance. Statistical significance was considered as *p* < 0.05. (Statistica 13.3, Statista).

## 4. Conclusions

The endothelium is easily accessible to drugs that circulate in the blood unless they are readily taken up by these cells. Therefore, anticancer drugs originally designed to interact with cancer cells, when intravenously injected, may interact with endothelial cells. Such an interaction can exert beneficial and adverse effects depending on the applied concentration. In this work, we showed that compounds of the type [Ru(dip)_2_L]^2+^ are moderately cytotoxic against HMEC-1 endothelial cells with IC_50_ at low micromolar concentrations. However, when low doses of these compounds are used, particularly Ru-bpy, they can induce marked changes in endothelial cells that inhibit their potency to angiogenesis. Ru-bpy inhibited cell mobility by increasing cell elasticity and modulating the expression of focal adhesion components. Such activity of Ru-bpy can be related to its significant effect on the disorganization of tube aggregation. Furthermore, the expression of ICAM-1 was elevated, while VCAM-1 decreased in endothelial cell protein extracts treated with Ru-bpy, supporting its negative impact on angiogenesis.

The presented results and our previous findings showing, among other effects, a significant impact on cancer cell adhesion and mobility [10,13,14,26], make the studied compounds interesting candidates as antimetastatic agents targeting various stages of metastasis. The studied compounds cause the desired effects at doses much lower than the cytotoxic ones, which may allow their application in less invasive low-dose therapy. It should be noted that the in vitro experiments used in the present study have many limitations, and in vivo studies are needed to support these findings. However, they can provide relevant information about the properties of the tested compounds that may deregulate or interfere with the metastasis process.

## Figures and Tables

**Table 1:** Cytotoxicity (IC_50_) of the studied Ru (II) complexes against HMEC-1 cells. Experiments were performed in triplicate and repeated three times. Results are presented as mean values and standard error of the mean.

Compound	IC_50_ [µM]
Ru-bpy	4.6 ± 1.0
Ru-NitroIm	6.9 ± 1.3
Ru-SC	5.1 ± 0.3

## Data Availability

The data presented in this study are available in the main text.

## Supplementary Materials

The following supporting information can be downloaded at: https://www.mdpi.com/article/10.3390/ijms23147708/s1.
