# Supplementary material for: Impact of Polypyridyl Ru Complexes on Angiogenesis—Contribution to Their Antimetastatic Activity
Source: Int J Mol Sci. 2022 Jul 12;23(14):7708. doi: 10.3390/ijms23147708 (PMC9323615; doi:10.3390/ijms23147708)

Supporting Information for

Impact of Polypyridyl Ru Complexes on Angiogenesis—Contribution to Their Antimetastatic Activity

Ilona Gurgul [1], Olga Mazuryk [1,*], Kamila Stachyra [2], Rafał Olszanecki [2], Małgorzata Lekka [3], Michał Łomzik [1,4], Franck Suzenet [5], Philippe C. Gros [6] and Małgorzata Brindell [1,*]

[1] Faculty of Chemistry, Jagiellonian University in Krakow, Gronostajowa 2, 30-387 Krakow, Poland; ilona.gurgul@uj.edu.pl (I.G.); michal.lomzik@chemia.uni.lodz.pl (M.Ł.)
[2] Chair of Pharmacology, Faculty of Medicine, Jagiellonian University Medical College, Grzegorzecka 16, 31-531 Krakow, Poland; kamila.stachyra@uj.edu.pl (K.S.) rafal.olszanecki@uj.edu.pl (R.O.)
[3] Department of Biophysical Microstructures, Institute of Nuclear Physics, Polish Academy of Sciences, PL-31342 Krakow, Poland; malgorzata.lekka@ifj.edu.pl
[4] Department of Organic Chemistry, Faculty of Chemistry, University of Łódź, ul. Tamka 12, 91-403 Łódź, Poland
[5] Institute of Organic and Analytical Chemistry, University of Orléans, UMR-CNRS 7311, rue de Chartres, BP 6759, CEDEX 2, 45067 Orléans, France; franck.suzenet@univ-orleans.fr
[6] Université de Lorraine, CNRS, L2CM, F-54000 Nancy, France; philippe.gros@univ-lorraine.fr
* Correspondence: olga.mazuryk@uj.edu.pl (O.M.); malgorzata.brindell@uj.edu.pl (M.B.)

Purity and identity confirmation

HPLC - HPLC-DAD Shimadzu LC-2030C, Shimadzu Europa GmbH, Duisburg, Germany

HRMS – micrOTOF-Q mass spectrometer, Bruker Daltonics, Billerica, MA, USA

1H NMR 400 MHz spectrometer, Bruker DRX400, Bruker Coperation, Billerica, MA, USA

Compound	HPLC+HRMS or NMR+HRMS analysis
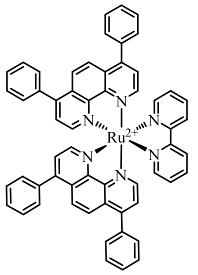	HRMS: m/z: Calculated for $[C_{70}H_{52}N_{10}ORu]$: m/z 458.1190, found 459.1188. 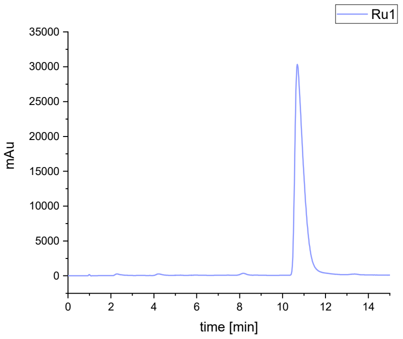
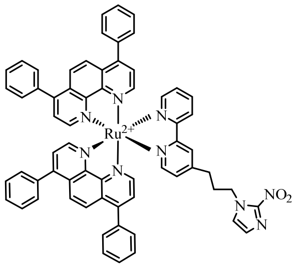	HRMS: m/z: Calculated for $[C_{64}H_{47}N_9O_2Ru]^{2+}$: m/z 537.6451 found: 537.6450 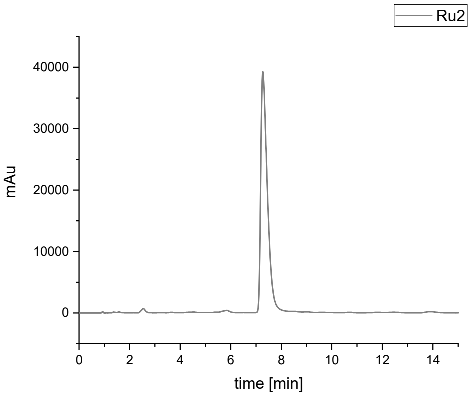

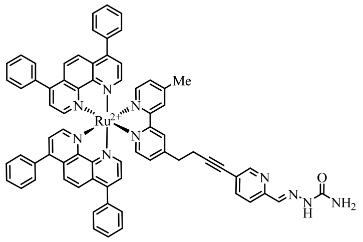

HRMS: m/z: Calculated for $[C_{70}H_{52}N_{10}ORu]^{2+}$: m/z 575.1679, found 575.1658.

^{1}H NMR (600 MHz, CD_3CN) δ 2.55 (s, 3H), 2.95 (t, *J* =6 Hz, 2H), 3.13 (t, *J*=6 Hz, 2H), 7.04 (s, 1H), 7.22 (d, *J*=6 Hz, 1H), 7.37 (dd, *J*=15.6 Hz, 6 Hz, 2H), 7.57-7.63 (m, 20H), 7.67 (d, *J*=5.4 Hz, 2H), 7.73 (dd, *J*=5.4 and 2.4 Hz, 2H), 7.79 (t, *J*=5.4 Hz, 1H), 8.13-8.20 (m, 6H), 8.23 (d, *J*=5.4 Hz, 1H), 8.26 (d, *J*=5.4 Hz, 1H), 8.33 (d, J=5.4 and 3.6 Hz, 1H), 8.80 (d, J=12.6 Hz, 1H), 8.90 (d, J=12.6 Hz, 1H) ppm

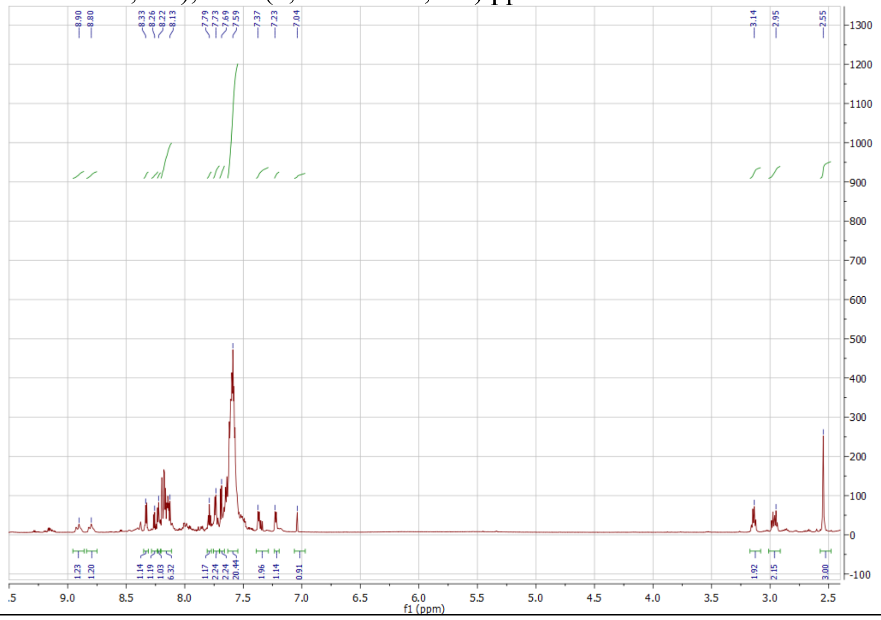

Supplement: Supplementary file 1 [file ijms-23-07708-s001.zip › ijms-1808443-supplementary.pdf]
